# Supplementary material for: Highly Dispersed Pt-Incorporated Mesoporous Fe2O3 for Low-Level Sensing of Formaldehyde Gas
Source: Nanomaterials (Basel). 2023 Feb 8;13(4):659. doi: 10.3390/nano13040659 (PMC9960270; doi:10.3390/nano13040659)
Supplement: Supplementary file 1 [file nanomaterials-13-00659-s001.zip › nanomaterials-2184959-supplementary.pdf]

nanomaterials

Supplementary Information

Highly Dispersed Pt-Incorporated Mesoporous Fe_2O_3 for Low-Level Sensing of Formaldehyde Gas

Seung Jin Jeon [1,2,†], Kyung Hee Oh [3,4,†], Youngbo Choi [2,*], Ji Chan Park [3,5,*] and Hyung Ju Park [1,*]

[1] Electronics and Telecommunications Research Institute (ETRI), 218 Gajeong-ro, Yuseong-gu, Daejeon 34129, Republic of Korea
[2] Department of Safety Engineering, Chungbuk National University, Cheongju 28644, Republic of Korea
[3] Clean Fuel Research Laboratory, Korea Institute of Energy Research, Daejeon 34129, Republic of Korea
[4] Department of Chemistry, Korea University, Seoul 02841, Republic of Korea
[5] Energy Engineering, University of Science and Technology, Daejeon 34113, Republic of Korea
* Correspondence: ybc@cbnu.ac.kr (Y.C.); jcpark@kier.re.kr (J.C.P.); park77@etri.re.kr (H.J.P.)
† These authors contributed equally to this work.

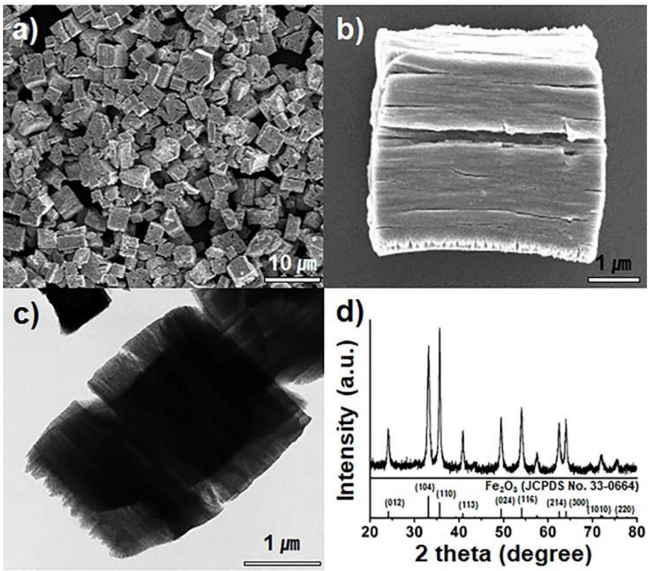

Figure S1. (**a**) SEM and (**b**) magnified SEM images, (**c**) TEM image, and (**d**) XRD spectrum of the m-Fe_2O_3.

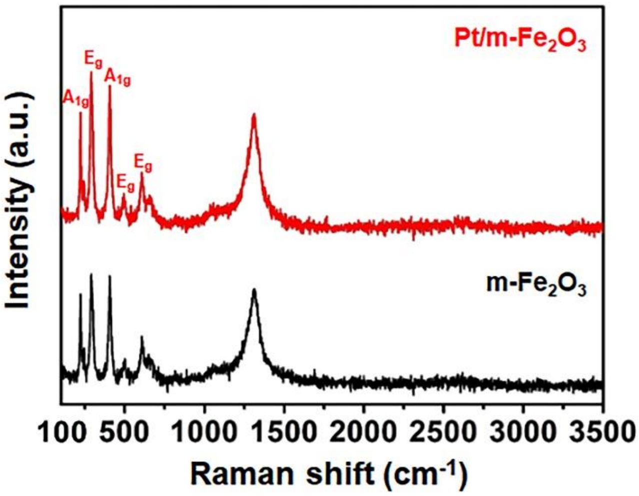

Figure S2. Raman spectra of the m-Fe_2O_3 and 0.2wt% Pt/m-Fe_2O_3.

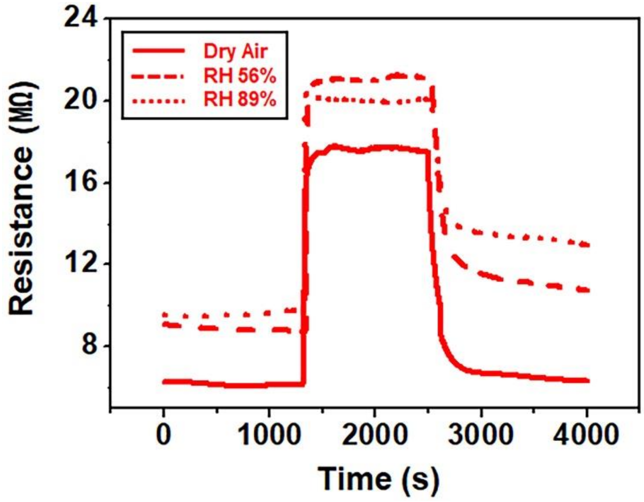

Figure S3. Resistance changes of the Pt/m-Fe_2O_3 under different relative humidity (Dry air, RH 56 % and RH 89 %) with respect to the 200 ppb HCHO gas at 300 °C.

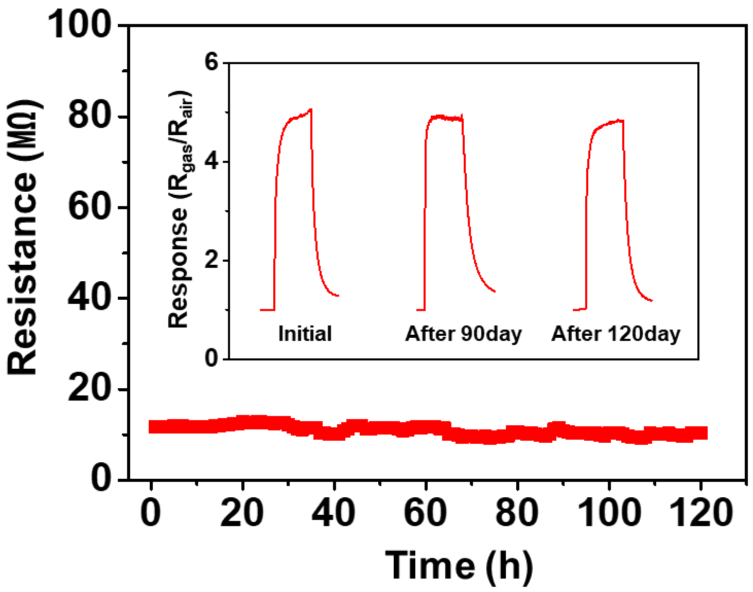

Figure S4. Base resistance of Pt-Fe_2O_3 in air condition for 120 h consecutively. (The insets were HCHO gas response of Pt-Fe_2O_3 toward 500 ppb HCHO gas at the initial test, after 90 days and 120 days at 275 °C).
